# Heterogeneous Catalytic Composites from Palladium Nanoparticles in Montmorillonite Intercalated with Poly (Vinyl Pyrrolidone) Chains

**DOI:** 10.3390/polym10060669

**Published:** 2018-06-15

**Authors:** Mengdie Xu, Jing Zhao, Guiqing Shu, Qi Liu, Minfeng Zeng

**Affiliations:** Zhejiang Key Laboratory of Alternative Technologies for Fine Chemicals Process, College of Chemistry and Chemical Engineering, Shaoxing University, Shaoxing 312000, China; joanne7280@163.com (M.X.); jingjingzhao218@sina.com (J.Z.); guiqingshu@163.com (G.S.); lq920819@163.com (Q.L.)

**Keywords:** poly (vinyl pyrrolidone), montmorillonite clays, Pd catalysis, catalytic composite, positron annihilation

## Abstract

In this study, poly (vinyl pyrrolidone) (PVP) chains intercalated montmorillonite (MMT) matrices has been demonstrated as an excellent scaffolding material for the immobilization of palladium (Pd) nanoparticles to prepare efficient heterogeneous catalysts for Heck reactions. Multiple layers (up to four) of PVP chains can intercalate the interlayer space of the MMT, resulting in an increase therein from 1.25 to 3.22 nm. MMT/PVP with PVP loading (20%) was selected as the platform for the immobilization of Pd. The in-situ reduction of the chelated Pd^2+^ into Pd^0^ in the interlayer space of MMT/PVP composite could be easily achieved. For the prepared Pd@MMT/PVP catalytic composite, a unique maze-like microstructure of Pd nanoparticles tightly encaged by PVP chains and by lamellae of layered silica has been detected by high resolution transmission electron microscopy (HR-TEM) and X-ray diffraction (XRD). Furthermore, the microstructure is well elucidated in molecular level by positron annihilation lifetime analysis of the Pd@MMT/PVP catalytic composite. The prepared Pd@MMT/PVP catalysts were highly active for the Heck coupling reactions between aromatic halides and alkenes, and could be recycled 9 times without significant decreases in coupling yields. The excellent comprehensive catalytic performances of the Pd@MMT/PVP catalytic composites are mainly attributed to their unique maze-like microstructure.

## 1. Introduction

Palladium (Pd) catalyzed Heck cross-coupling reactions are among of the most versatile methods for the construction of C–C bonds linked to unsaturated double bonds in organic synthesis [1,2,3]. Nowadays, the Heck reaction is widely used for the synthesis of many natural products, biologically active compounds, fine chemicals, and their intermediates with alkene structure units [4,5,6,7,8,9]. Great attention has been given to the development of novel and advantageous catalytic systems for Pd-catalyzed Heck reactions [10,11,12]. Among them, homogeneous Pd-based catalysts are preferred due to their excellent catalytic activity and selectivity. However, the homogeneous catalysts for Heck reactions often have present the problems of difficult separation, recovery, easy deactivation, and high costs, which greatly limit their wide applications in industry. Such drawbacks can be effectively reduced in heterogeneous catalysis processes. On the one hand, the immobilization of Pd nanoparticles on a support can significantly reduce the aggregation of the nanoparticles to prevent the loss of the activity. On the other hand, the heterogeneously supported Pd catalysts can easily be separated from the reaction system and recycled to the next reaction. Therefore, preparations of heterogeneously supported catalysts by immobilization of Pd species on various supports with excellent recovery performance have become the focus of the catalytic system for Heck reactions [13,14,15].

Many solid supporting materials, such as carbon materials [16,17], silica [18,19], TiO_2_ [20,21], Fe_3_O_4_ [22,23], clay [24,25], polymers [26,27,28], and so on, have been reported as palladium supports applied in Heck reactions. Among them, a type of natural layered silicate clay of montmorillonite (MMT) which contains negatively charged layers and interlayers with metal cations, like Na^+^, Ca^2+^, etc., are often used as transition metal catalyst supports. The transition metal cations can be easily introduced into the interlayers of MMT by an ion-exchange process for high cation exchange capacity. In addition, MMT has remarkable stability against mechanical agitations, good swelling capacities, strong adsorption properties, and high thermal stabilities. Kaneda et al. [29] synthesized sub-nano ordered Pd clusters within the interlayer space of MMT, and the prepared Pd-MMT heterogeneous catalyst showed high catalytic activity for allylic substitution reactions. Dutta et al. [30] also prepared a Pd-MMT heterogeneous catalyst by the introduction of Pd^0^-nanoparticles into the nanopores of sulfuric acid modified MMT, which showed excellent activity when applied in a Heck reaction and a Sonogashira reaction. However, as an inorganic support, although there are a number of hydroxyl groups located on the surface of the silicate layer, the chelation capability of MMT to transition metals which encaged in the interlayer space is generally not strong enough, and the recyclable times of such a Pd-MMT heterogeneous catalyst is limited (3 times [30]). This indicates that MMT needs further modification to enlarge the interlayer space and improve its interactions with the encaged transition metal species. Cationic surfactants, such as cetyl trimthyl ammonium bromide (CTAB), are often used as organic modifiers for MMT. For example, Zhou et al. [31] reported that Ru nanoparticles supported on CTAB modified MMT supports showed high activity and selectivity in the hydrogenation of quinolone reactions. Recently, we have succeeded in preparing a high stable Pd heterogeneous catalyst for coupling reactions by encaging Pd^0^ nanoparticles within a cationic polymer of chitosan (CS) modified MMT supports [32]. On the one hand, the intercalation of CS is beneficial for expanding the interlayer space of MMT from 1.25 nm (MMT) to 1.97 nm (Pd@MMT/CS), which is advantageous for the reaction substrates to easily access the entrapped Pd species. On the other hand, CS chains have strong chelation capabilities with Pd species, which is good for the tight encaging of the Pd species within the interlayer space of MMT. It revealed that the construction compatible inorganic MMT/polar polymer composite supports to immobilize metal catalysts is a good solution for achieving satisfactory comprehensive properties.

Besides CS, other nonionic polymers, such as poly (vinyl pyrrolidone) (PVP) can also effectively intercalate into the MMT interlayer using a similar solution intercalation method [33]. PVP is one of the most used polymer stabilizers for transition metal nanoparticles to prevent aggregation into big-sized particles [34,35,36]. The amide groups within the macromolecular backbone of PVP have good chelation capabilities with transition metals. Furthermore, PVP itself is demonstrated as an excellent polymer support for transition metals in preparing heterogeneous catalysts [37,38]. For example, Bai et al. reported [37] a novel catalyst containing Pd nanoparticles supported on PVP nanofiber films, showing fairly good efficiency in hydrogenation reactions of nitro compounds and Heck reactions. However, as a polymer support like CS, PVP support has limited stability against mechanical agitations, heat, and solvents, etc. The Pd@PVP heterogeneous catalyst prepared by Bai et al. can be only recycled 3 times. Drawing from the above works, we believe that heterogeneous catalysts with excellent comprehensive properties could be also prepared by the immobilization of Pd nanoparticles in the interlayer space of a PVP intercalated MMT composite. Previously, Dekany et al. [39,40,41] carried out some preliminary examinations of the influences of the preparation process conditions on the size of metal particles formed on the layer surface and interlayer space of MMT intercalated with PVP. The results showed that the average size of transition metal nanoparticles can be kept within 1–6 nm. However, application of the novel Pd nanoparticles supported on PVP intercalated MMT composites in catalysis is still lacking, which motivated us to systemically explore the microstructure and catalytic performance of this novel, heterogeneous catalytic composite.

**Scheme 1:**
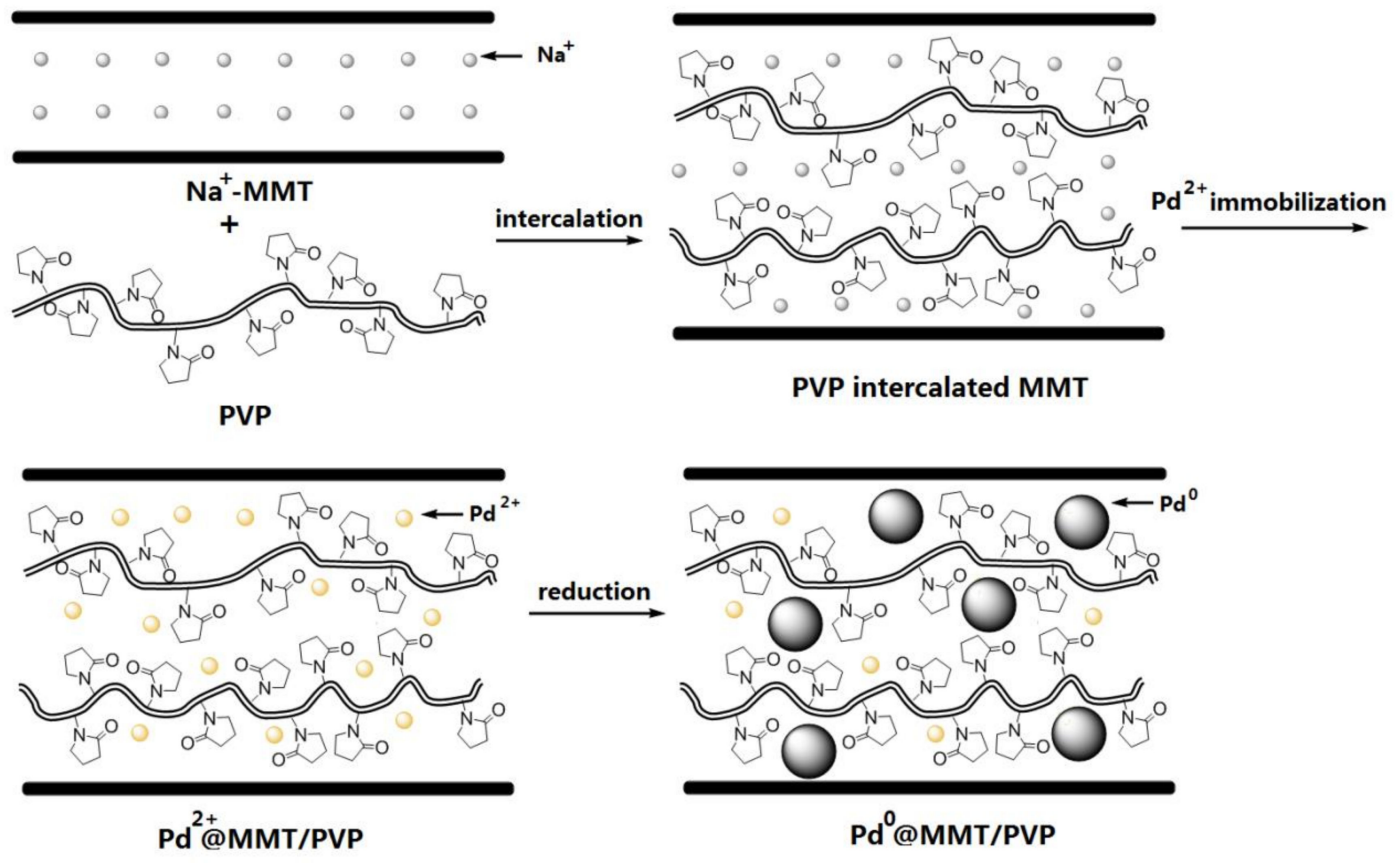
Process of the preparation of the Pd@MMT/PVP catalytic composite.

In this study, the preparation process of the novel Pd^0^@MMT/PVP catalytic composites can be illustrated in Scheme 1, which includes three steps: (i) intercalation of PVP into Na^+^-MMT by interactions with hydroxyl groups on the surface of MMT and exchanges with the solvents molecules previously diffused in MMT, (ii) immobilization of Pd^2+^ on the MMT/PVP composite by cations exchanges of Pd^2+^ with Na^+^ and chelation with PVP chains, (iii) in situ reduction of Pd^2+^ into Pd^0^ encaged in the interlayer space of PVP intercalated MMT. The unique microstructure of the prepared Pd^0^@MMT/PVP catalysts has been characterized by several methods, including X-ray diffraction (XRD), X-ray photo electron spectroscopy (XPS), high resolution transmission electron microscopy (HR-TEM), thermal gravity analysis (TGA), and inductively coupled plasma-atomic emission spectroscopy (ICP). Moreover, positron annihilation lifetime spectroscopy (PALS) is further used to elucidate the sub-nano level microstructure of Pd@MMT/PVP catalytic composites. The catalytic performances of the Pd@MMT/PVP catalytic composite catalyzed Heck reaction in DMSO/ethylene glycol mixed solution were investigated. 

## 2. Materials and Methods 

### 2.1. Materials

G-105 type of Na^+^−MMT was obtained from Nanocor Co., Arlington Hts, IL, USA (cationic exchange capacity: 145 mmol/100 g). K-30 type of PVP was obtained from Sinopharm Chemical Reagent Co., Ltd., Shanghai, China (*M*_n_ = 4 × 10^4^). PdCl_2_ was obtained from Zhejiang Metallurgical Research Institute Co., Ltd., Zhejiang, China. The Heck reaction substrates of aromatic halides, acrylates and alkenes were obtained from Energy Chemical, Sun Chemical Technology (Shanghai) Co., Ltd., Shanghai, China. Other chemical reagents and solvents in analytical purity grade were obtained from Sinopharm Chemical Reagent Co., Ltd., Shanghai, China.

### 2.2. Preparation of MMT/PVP Supports and Pd@MMT/PVP Catalytic Composite

A homogeneous suspension solution was prepared by dispersing 2 g of MMT into 100 mL of deionized water under magnetic stirring. A specific amount of PVP was dissolved in 100 mL acetic acid solution (2 wt %). The PVP solution was then added into the MMT suspension solution and stirred magnetically at 55 °C for 12 h. The mass ratios of MMT/PVP were 100/0, 90/10, 80/20, 70/30, 60/40, 50/50. Then, 2 mL of Pd^2+^ solution (0.3 g of PdCl_2_ and 2 g of NaCl co-dissolved in 100 mL deionized water) was added drop-wise into the MMT/PVP (80/20) mixture and stirred for another 6 h. The Pd^2+^@MMT/PVP composite was centrifugally separated and washed repeatedly with deionized water to neutralize (pH = 7). The resultant Pd^2+^@MMT/PVP composite was dried at 60 °C. Before catalysis use, Pd^2+^@MMT/PVP composite was reduced to Pd^0^@MMT/PVP catalytic composite by ethylene glycol at 80 °C for 30 min. The effect of reduction with alcohols is similar to that with hydrogen gas [42]. ICP-AES determination of Pd content in the Pd@MMT/PVP catalyst showed the content of the metal was about 0.2 wt %.

### 2.3. Characterizations of the MMT/PVP Supports and Pd@MMT/PVP Catalytic Composite

The crystalline structure of the MMT/PVP supports and Pd@MMT/PVP catalytic composite were measured with an Empyrean X-ray diffraction system (PANalytical, Eindhoven, the Netherlands). The recorded 2θ range was 2–70° and the scanning rate was 2°/min. The thermal stabilities of MMT/PVP supports and Pd@MMT/PVP catalytic composites were measured with a Mettler Toledo TGA/DSC 2 STAR system. The temperature range was 30–700 °C and the heating rate was 20 °C/min at air atmosphere. The XPS analysis of the binding energy of Pd elements within Pd@MMT/PVP catalyst was measured with a Thermos Scientific ESCALAB 250 Xi X-ray photoelectron spectrometer. The morphology of Pd@MMT/PVP catalytic composite was measured with a JEM-2100F HR-TEM (JEOL Ltd., Tokyo, Japan). The contents of Pd in the Pd@MMT/PVP catalytic composite were determined by Leemann ICP-AES Prodigy XP inductively coupled plasma atomic emission spectrometry. The PALS analysis of MMT/PVP supports and Pd@MMT/PVP catalysts was performed with an EG & G ORTEC fast-slow system. The time resolution of the PALS analysis was about 198 ps. The samples of MMT/PVP supports and Pd@MMT/PVP catalysts were pressured into thin disks firstly (diameter × thickness: 1 cm × 2 mm). The positron source (^22^ NaCl, 16 μCi) was deposited between two Kapton foils. This was then sandwiched between two pre-pressured samples disks. To obtain good statistics, the total counts of the lifetime spectra of each sample are at least 2 × 10^6^. The positron annihilation spectra were analyzed by Lifetime9.0 and MELT-4 (Maximum Entropy for Lifetime Analysis-4) program.

### 2.4. Catalytic Performance of the Pd@MMT/PVP Catalytic Composite

In a 50 mL round bottom flask, a mixture of aromatic halide (1 mmol), acrylates or alkenes (2 mmol), Pd^0^@MMT/PVP catalyst (3 μmol of Pd), CH_3_COOK (3 mmol), and solvent (5 mL DMSO + 0.2 mL ethylene glycol) was stirred magnetically at 110 °C (heated in oil bath) for 5 h. The reaction progress was detected with the layer chromatography (TLC) method, and a gas chromatography-mass spectrometry (GC/MS) analysis was performed. The Heck coupling yield was obtained from a GC/MS quantitative analysis of the reaction mixture. The chemical structure of all products was confirmed by H^1^ NMR and GC/MS analysis, which is consistent with our recent work [32]. In the recycling experiment, the Pd@MMT/PVP catalytic composite was filtrated out from the reaction system after each reaction run, repeatedly washed with ethanol 3–5 times, and then reused for the next reaction run. 

## 3. Results and Discussion

**Figure 1:**
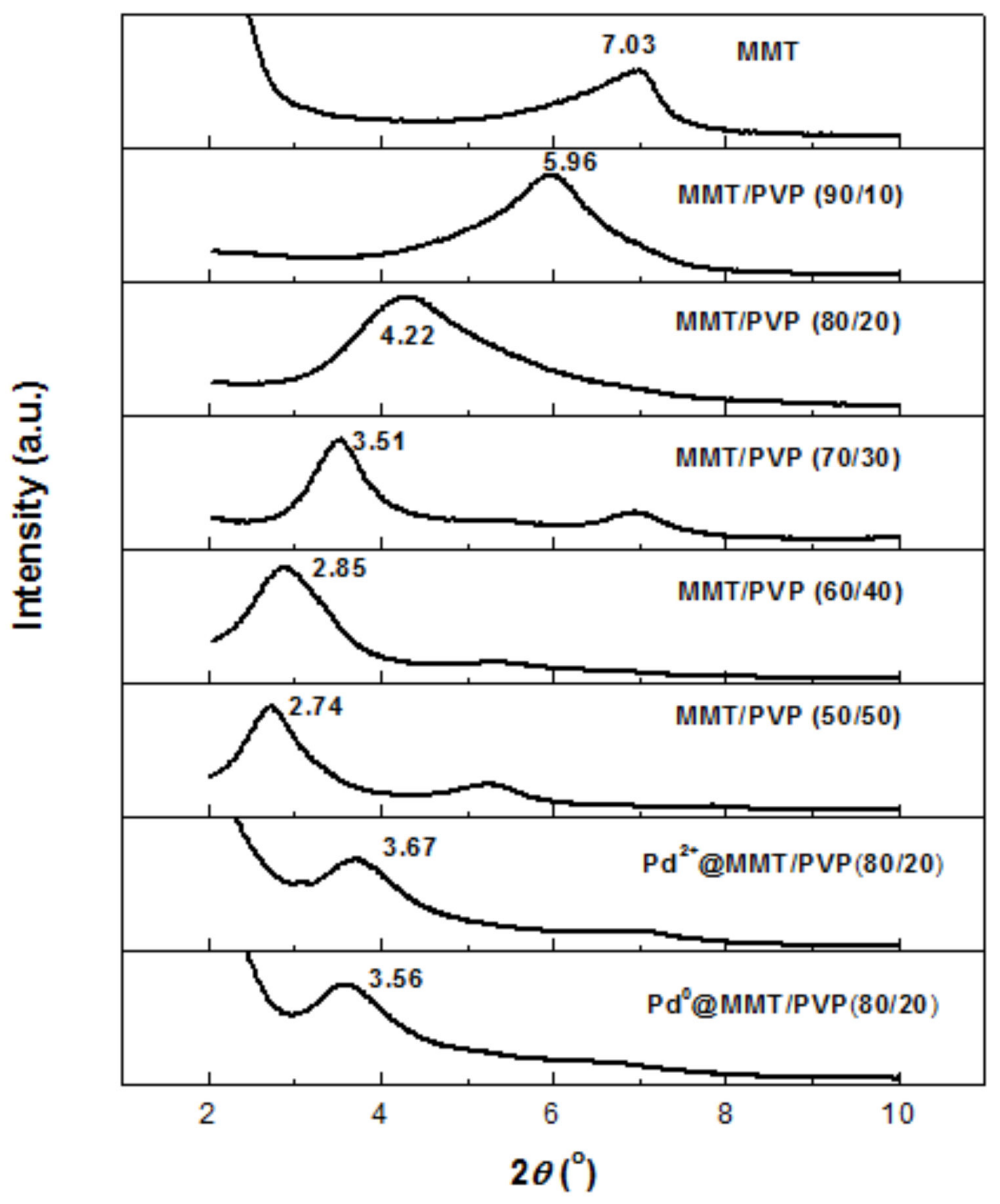
XRD patterns of MMT, MMT/PVP support and Pd@MMT/PVP catalytic composite.

Figure 1 compares the X-ray diffraction patterns from pure MMT, composites of MMT/PVP with different PVP loading, and Pd@MMT/PVP catalytic composites with reduction treatment. The characteristic reflection peak related to the basal spacing of *d*_001_ of pure MMT is found to be 2θ of 7.03° (Figure 1), indicating the typical layer-to-layer distance of pure MMT is 1.25 nm from Bragg equation [33]. This reflection peak shifts to lower angles as the PVP polymer loading increases, i.e., 5.96° (10% loading), 4.22° (20% loading), 3.51° (30% loading), 2.85° (40% loading), 2.74° (50% loading), respectively. The corresponding layer-to-layer distances were calculated to be 1.48 nm (10% loading), 2.09 nm (20% loading), 2.51 nm (30% loading), 3.10 nm (40% loading), 3.22 nm (50% loading). This reveals that the intercalation of PVP polymers in the interlayer space of MMT leads to an efficient expansion of the interlayer distance. 

As a kind of highly polar polymer, PVP macromolecules can form strong interactions with –OH groups on the surface of MMT layers, which is the main driving force for PVP macromolecules intercalation into the interlayer space of MMT in solution. During the intercalation process, the PVP chains preferentially take extended conformation in order to maximize their interactions with the MMT layers. Taking into account the thickness of the silica layer (about 0.96 nm) [33,43] and the extended PVP chain (about 0.52 nm) [44,45], the increased interlayer spacing of MMT/PVP (90/10) (*d*_001_ of 1.48 nm, 0.52 nm larger than MMT) is attributed to the formation of intercalation of mono-layer of PVP chain into the interlayer space of MMT. Increasing PVP loading results in the formation of intercalation of double layers of PVP chains into MMT, as shown in the diffraction result on MMT/PVP composite with 20 wt % PVP loading (*d*_001_ of 2.09 nm, 1.03 nm larger than MMT). It was found that the number of the intercalated PVP chains layers could be further increased with an increase of PVP loading. In the case of MMT/PVP (70/30), the *d*_001_ spacing is 2.51 nm (1.55 nm larger than MMT), indicating three layers of PVP chains have intercalated into the MMT. Similarly, four layers of PVP chains have intercalated into the MMT in the case of the MMT/PVP composite with 40 wt % PVP loading (*d*_001_ of 3.10 nm, 2.14 nm larger than MMT). This is difficult in the intercalation of more than four layers of PVP chains in MMT, as the interlayer spacing increased limitedly when PVP loading is 50% (*d*_001_ of 3.22 nm, 2.26 nm larger than MMT).

**Figure 2:**
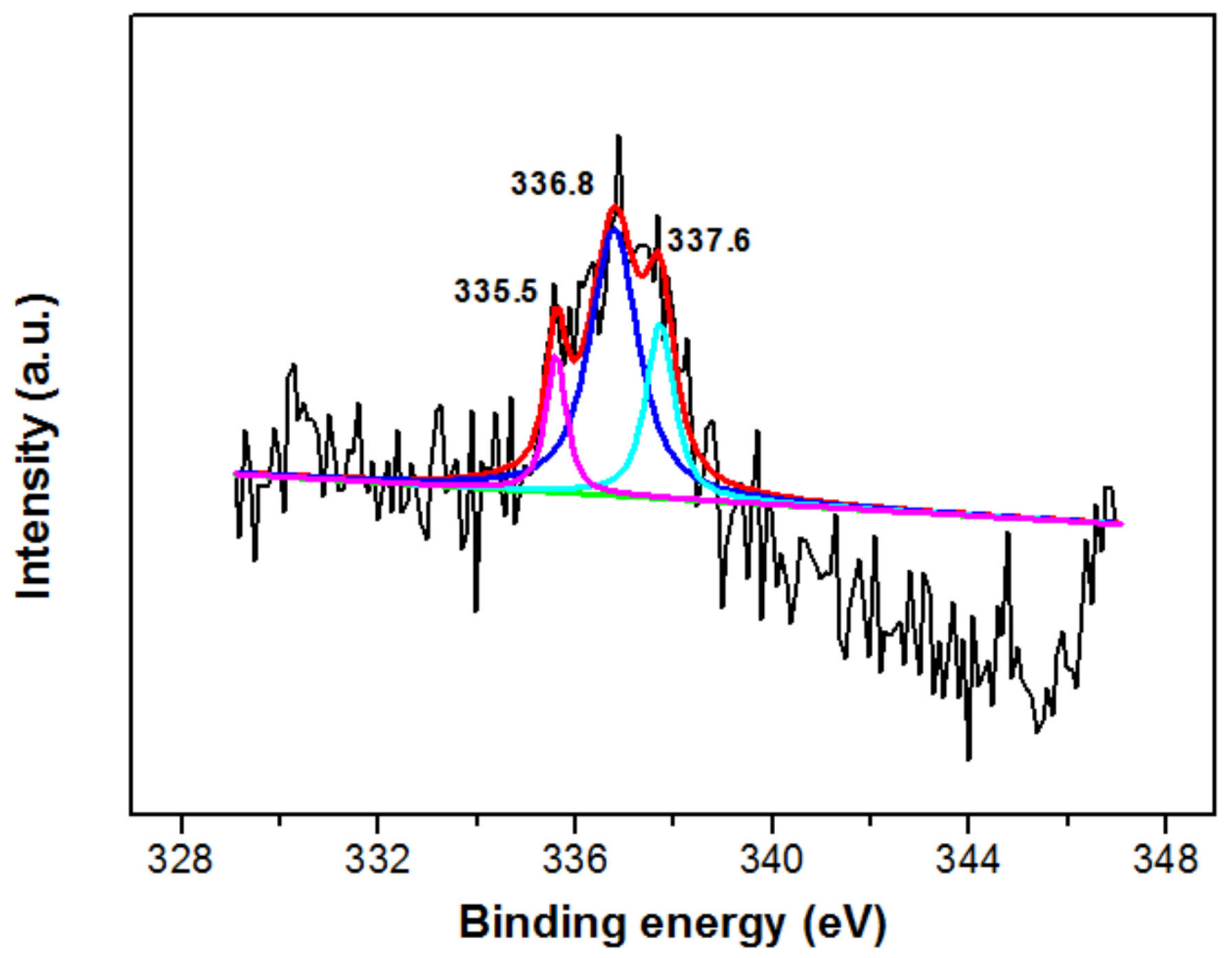
XPS spectra of the Pd^0^@MMT/PVP catalytic composite.

Too much PVP content in the MMT/PVP composite could be disadvantageous for high physical properties (such as thermal stability, mechanical properties, and resistance to solvents etc.) because of the lower stability of PVP than MMT. In addition, too much PVP content might also lead to an exfoliation structure of MMT layers; the stabilization effects of both MMT and PVP on the Pd nanoparticles would then decrease significantly. Therefore, we chose MMT/PVP with a moderate PVP loading (20%) as the platform for immobilization of Pd. As shown in Figure 1, after chelation of Pd^2+^ with the amide groups of PVP that intercalated in MMT, the interlayer spacing increases from 2.09 nm (MMT/PVP (80/20)) to 2.40 nm (Pd^2+^@MMT/PVP (80/20)). The immobilized Pd^2+^ can be reduced by ethylene glycol. According to the XPS analysis results (Figure 2), it is evidenced that both Pd^2+^ (characteristic Pd 3d_5/2_ electron binding energy at 337.6 eV and 336.8 eV) and Pd^0^ (characteristic Pd 3d_5/2_ electron binding energy at 335.5 eV) species co-exist in the Pd^0^@MMT/PVP (80/20) composite [46]. The interlayer spacing of Pd^0^@MMT/PVP (80/20) composite further increases to 2.48 nm, which should be caused by local distortions of the MMT layered structures by the in-situ generation of Pd^0^ nanoparticles.

**Figure 3:**
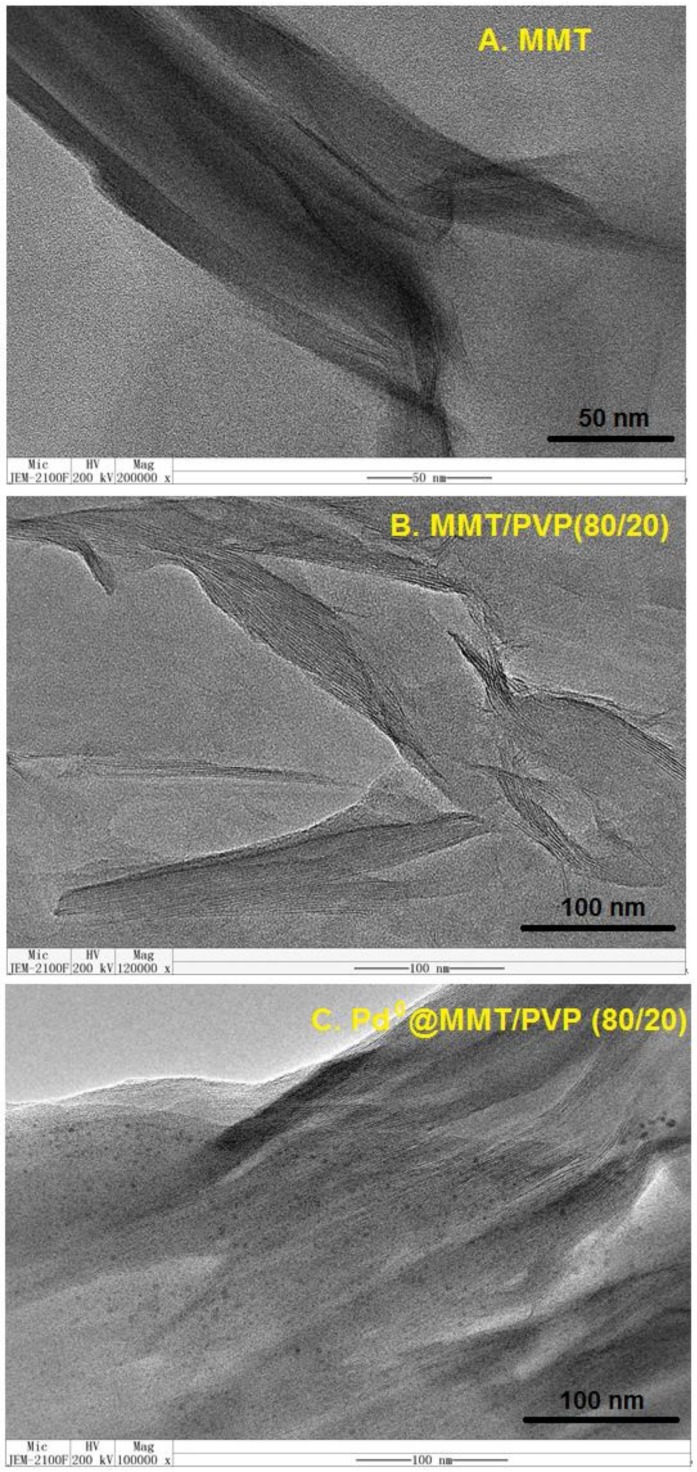
HR-TEM images of MMT (**A**), MMT/PVP support (**B**) and Pd@MMT/PVP catalytic composite (**C**).

Figure 3 shows the morphology of pure MMT, MMT/PVP, and Pd^0^@MMT/PVP catalytic composite, as observed by HR-TEM. MMT shows a two-dimensional porous layer structure, but the layer spacing is small and the interlayer contract is not clear, as visualized by HR-TEM. After the intercalation of PVP chains, the interlayer spacing of MMT increases significantly. The interlayer contract then becomes much clearer than that of MMT. After immobilization of Pd^2+^ and reduction with ethylene glycol, many Pd^0^ nanoparticles can be visualized in the MMT/PVP matrices. The dispersed Pd^0^ nanoparticles are mainly in three forms: (i) Pd^0^ nanoparticles sized 3–5 nm clipped in 2–3 layers of MMT, (ii) Pd^0^ nanoparticles sized 1–2 nm dispersed in the interlayer space of MMT, which show up as dark bands between neighboring galleries of the image, (iii) Pd^0^ nanoparticles sized 3–5 nm dispersed on the surface of MMT layer. The microstructure in the interlayer space of the Pd^0^@MMT/PVP catalytic composite is much like a “maze”, in which the intercalated PVP chains and encaged Pd species acts as the “hedges”. Such a maze-like microstructure might be responsible for good catalytic performance. On the one hand, the much expanded interlayer space of MMT by multi layers of PVP intercalation makes the diffusion of reactant substrates into the “maze” much easier. On the other hand, the encaged Pd species is difficult to leach out to the reaction solution from the “maze”.

To obtain a good understanding of the microstructure of MMT/PVP matrices and Pd@MMT/PVP catalytic composites in molecular level, PALS was used as a testing method to provide information on the micro defects of the composite [47,48,49]. In molecular solids, a part of the implanted positrons (e^+^) will combine with electrons (e^−^) of the surrounding molecules to form positroniums. According to the different electron and positron spin states, positroniums are classified into two types: para-positronium (*p*-Ps, singlet spin state), and ortho-positronium (*o*-Ps, triplet spin). Generally, three positron annihilation processes are possible, i.e., *p*-Ps annihilation (shortest lifetime component, τ_1_), free positron annihilation (intermediate lifetime component, *τ*_2_), and *o*-Ps annihilation (longest lifetime component, *τ*_3_). The mean diameter of micro-defects within composite *D* can be calculated from the lifetime *τ*_3_ through Equations (1) and (2), according to the Tao-Eldrup free volume model [50,51]. Where Δ*R* = 0.1656 nm is the fitted empirical electron layer thickness.

(1) $$\frac{1}{\tau_{3}}=2\{1-\frac{R}{R+\Delta R}+\frac{1}{2\pi }\sin(\frac{2\pi R}{R+\Delta R})\}$$

*D* = 2*R* (2)

As shown in Table 1, the PALS spectra was fitted well in three-component lifetimes, *p*-Ps annihilation (*τ*_1_, and its intensity, *I*_1_), free positron annihilation (*τ*_2_, and its intensity, *I*_2_), *o*-Ps annihilation in the micro-defects of MMT/PVP and/or Pd@MMT/PVP composites (*τ*_3_, and its intensity, *I*_3_). For pure MMT, the *o*-Ps annihilation lifetime is 2.806 ns, attributing to the *o*-Ps annihilation in the interlayer space of MMT. The *D* value can be calculated to be 0.6988 nm from Equations (1) and (2). After intercalation with mono layer of PVP chains, *o*-Ps annihilation mainly occurs in the micro defects between the intercalated PVP mono layer and MMT layer, and the *D* value of MMT/PVP (90/10) is 0.6072 nm. This reveals that the size of the micro defects obtained by PALS decreases though the interlayer space between MMT interlayers has been expanded by the intercalated mono layer of PVP chain. After intercalation of 2–4 layers of PVP chains, *o*-Ps annihilation will occur in the micro defects not only in the space between the intercalated PVP chains and MMT layers, but also in the space between intercalated PVP chains themselves. Therefore, a similar trend of a slight decrease in *D* value is observed as PVP loading increases. The changes in the microstructure after immobilization of Pd species are sensitively detected by PALS. After immobilization of Pd^2+^, the *D* value increases from 0.6062 nm (MMT/PVP (80/20)) to 0.6158 nm (Pd^2+^@MMT/PVP (80/20)). For the chelation of Pd^2+^ cations with amide groups of PVP chains, they are preferred to be entrapped between the PVP chain layers. Over all, the interlayer spacing is further expanded (also detected in XRD measurement), which leads to an increase of the size of all the micro defects. The effects of the reduction of Pd^2+^ to Pd^0^ on the changes of the microstructure are sensitively detected by PALS, too. As shown in Table 1, the *D* value increases from 0.6158 nm (Pd^2+^@MMT/PVP (80/20)) to 0.6208 nm (Pd^0^@MMT/PVP (80/20)). Usually, the Pd^0^ species has poorer chelation capability with PVP chains than Pd^2+^ species. Therefore, the compactness of the PVP chains will undergo a decrease after the reduction of Pd^2+^ to Pd^0^, leading to an increase in the size of micro defects. In addition, it is likely that the interlayer spacing enlargement caused by the formation of large-sized Pd^0^ nanoparticles with distorted microstructures is another reason for this increase in the size of micro defects. 

**Figure 4:**
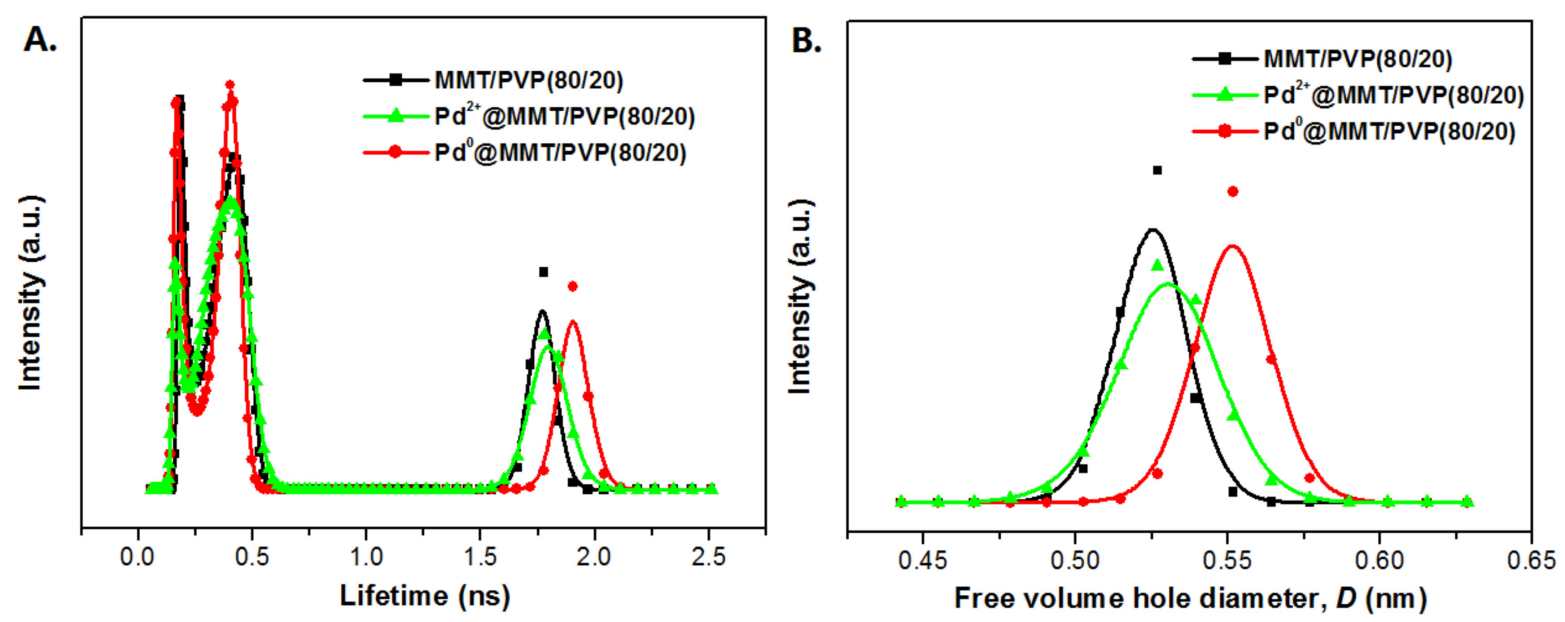
The distribution of positron annihilation lifetime (**A**) and calculated micro defects size (**B**) of MMT/PVP support, Pd^2+^@MMT/PVP and Pd^0^@MMT/PVP catalytic composite.

The PALS spectra of the Pd@MMT/PVP composites are further analyzed by MELT-4 program to obtain the distribution of the lifetimes. As shown in Figure 4A, there are three lifetime peaks for each sample and the longest lifetime peak is attributed to the *o*-Ps annihilation of the composites. For MMT/PVP (80/20), the *o*-Ps lifetime ranges from 1.55 ns to 1.98 ns. After immobilization of Pd^2+^, the *o*-Ps lifetime shifts to longer lifetime range (1.55–2.05 ns). Reduction of Pd^2+^ to Pd^0^ leads a further longer shift of the *o*-Ps lifetime (1.65–2.15 ns). The size distribution of the micro defects as calculated from Equation1 and Equation2 of MMT/PVP (0.4786–0.5644 nm), Pd^2+^@MMT/PVP (0.4780–0.5770 nm), and Pd^0^@MMT/PVP (0.5148–0.6026 nm) is shown in Figure 4B. Clearly, the variances trend of size distribution by MELT analysis corresponds with that of mean size by LT analysis. In summary, the microenvironment results based on PALS analysis provides strong evidence that both the extensive PVP chain layers and most of the Pd species are well incorporated into the expanded interlayer space of MMT.

**Figure 5:**
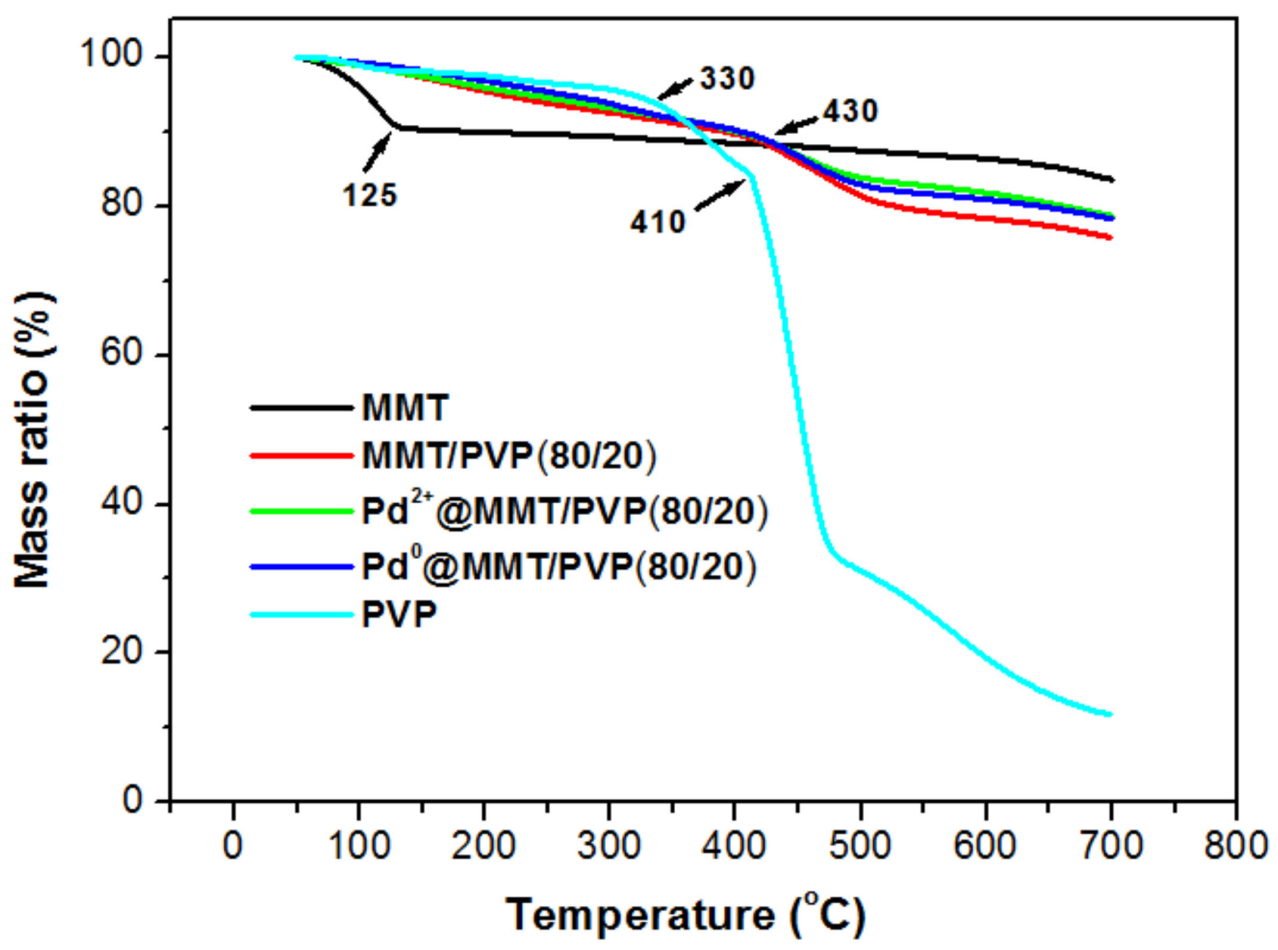
TGA curves of MMT, PVP and Pd@MMT/PVP catalytic composite.

The thermal stabilities of pure MMT, PVP, MMT/PVP (80/20), and Pd@MMT/CS (80/20) catalyst were measured with TGA (Figure 5). MMT shows excellent thermal stability, except during a weight loss stage before 125 °C, which was attributed to the evaporation of absorbed and/or bonded H_2_O. The first weight loss stage of PVP is observed from 330 to 410 °C. Then, PVP show serious decomposition of PVP chains at higher temperature than 410 °C. The thermal degradation curves of MMT/PVP, Pd^2+^@MMT/PVP, and Pd^0^@MMT/PVP are almost overlapped, indicating similar thermal stabilities with each other. In these three cases, the starting decomposition temperature of PVP component in the composites is improved to 430 °C, exhibiting much higher thermal stability than pure PVP after intercalation into the MMT layers. The high thermal stability of the Pd@MMT/PVP catalytic composite should be advantageous for good recyclability.

The catalytic activities of the Pd^0^@MMT/PVP were explored in the Heck coupling reactions of aromatic halides with acrylates or alkenes in DMSO solution. As shown in Table 2, the novel Pd^0^@MMT/PVP catalyst shows high catalytic activity for the reaction iodo benzene with n-butyl acrylate (entry 1) with a yield of 98%. It also shows high catalytic efficiencies for the substituted aryl iodides having an electron-donating group like *p*-CH_3_ (entry 2, 93% yield), *o*-CH_3_ (entry 3, 89% yield), *m*-OCH_3_ (entry 4, 87% yield), or having an electron-withdrawing group such as *p*-F (entry 5, 90% yield), *m*-F (entry 6, 89% yield), and *o*-Cl (entry 7, 87% yield). The coupling reactions of substituted iodo benzene with styrene can be also well catalyzed by the Pd^0^@MMT/PVP catalyst (entry 8–10). These catalysis application results indicate that all the reactant substrates can easily diffuse into the interlayer space of MMT to get access to the entrapped Pd species. For a higher bond strength of C-Br than C-I bonding, the Pd^0^@MMT/PVP catalyst has low catalytic activity for the coupling reaction of bromo benzene with n-butyl acrylate and/or styrene (entries 11, 12, trace yield). Nevertheless, C-Br bond can be effectively activated with an electron-withdrawing group such as the *m*-COCH_3_ group (entries 13, 14, moderate yields close to 50%). Such Pd^0^@MMT/PVP catalysts have similar high catalytic activities with most of other recently reported Pd heterogeneous catalysts, such as Pd@PVP nanofiber membrane catalyst [37], Pd@MMT catalyst [30,31], and Pd@MMT/CS catalyst [32], etc.

**Figure 6:**
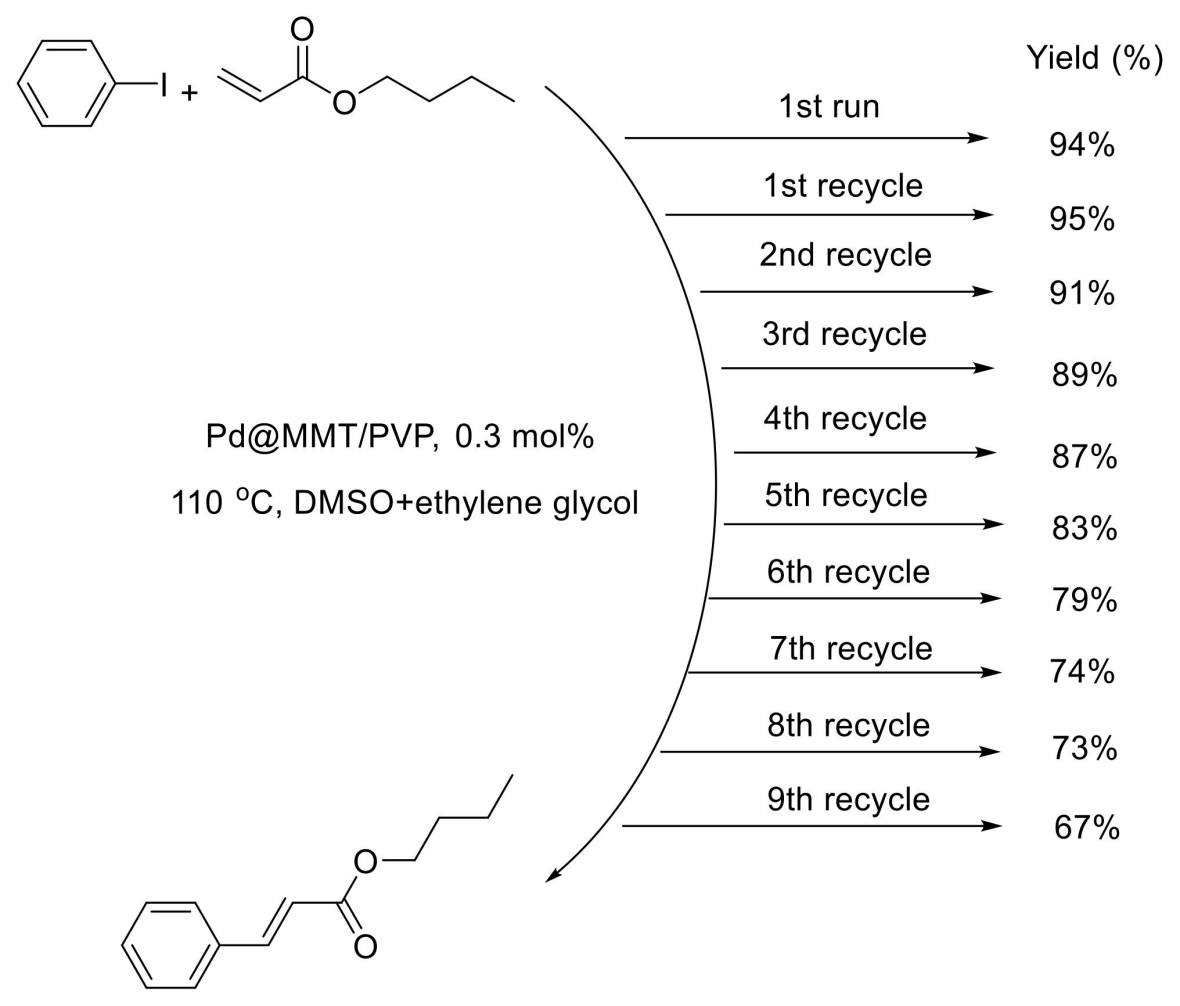
Dependence of the Heck reaction yield on the recycle times of the Pd^0^@MMT/PVP catalytic composite.

As shown in Figure 6, the stability and reusability of Pd^0^@MMT/PVP catalyst were assessed with a model reaction of iodo benzene coupling with n-butyl acrylate. During the recycling experiments, the Pd^0^@MMT/PVP catalyst could easily be filtrated out from the reaction system for use in the next reaction run. An ICP analysis of the reaction solution after filtration confirmed that the Pd content was below the detection limit, indicating that Pd nanoparticles are tightly entrapped within the interlayer space of MMT/PVP supports. Moreover, 67% yield for the recycled Pd^0^MMT/PVP catalyst catalyzed Heck reaction after 9 cycles was observed. Clearly, the recyclable times of Pd^0^@MMT/PVP catalyst are fewer than that of recent reported Pd^0^@MMT/CS catalyst (30 times) [32], which is mainly due to extremely high chelation capacities of CS with Pd species. Nevertheless, with similar Pd loading amounts, the Pd^0^@MMT/PVP catalyst has much higher recyclability than many of other Pd heterogeneous catalysts, such as Pd@PVP nanofiber films (3 times) [37], and/or Pd@MMT catalysts (3 times) [30,31], etc. This indicates that the unique maze-like microstructure of the Pd^0^@MMT/PVP catalyst is advantageous for the slow leaching of the Pd species. 

## 4. Conclusions

In this study, it was demonstrated that the intercalation of multi layers of PVP chains into Na^+^-MMT provides novel MMT/PVP matrices for immobilization of Pd species. The prepared Pd^0^@MMT/PVP catalysts show fairly good catalytic activities for Heck reactions, and can be recycled 9 times with high yields. The excellent comprehensive catalytic performances of the Pd@MMT/PVP catalysts are mainly attributed to their unique maze-like microstructure, which is well elucidated by positron annihilation lifetime spectrum and other methods. On the one hand, the reactant substrates can easily diffuse into the enlarged interlayer space of MMT to get access to the tightly entrapped Pd species. On the other, a perfect combination of the excellent stabilizing of PVP for Pd nanoparticles and remarkable stability of MMT was achieved in the well-designed Pd^0^@MMT/PVP catalytic composite. Such a novel catalyst overcomes the shortages of Pd leaching and difficult Pd recovery in conventional Pd homogeneous catalysis, and shows a broad foreground in both experimental and industrial applications. 

## Figures and Tables

**Table 1:** Variation of positron annihilation lifetimes, intensities, and mean diameter of micro-defects of the MMT, MMT/PVP support and Pd@MMT/PVP catalytic composite analyzed by LT-9 program.

Sample	*τ*_1_ (ns)	*I*_1_ (%)	*τ*_2_ (ns)	*I*_2_ (%)	*τ*_3_ (ns)	*I*_3_ (%)	*D* (nm)
MMT	0.2356	63.7	0.5125	33.2	2.806	3.1	0.6988
MMT/PVP (90/10)	0.1977	51.6	0.449	43.3	2.213	5.1	0.6072
MMT/PVP (80/20)	0.2025	57.3	0.4755	36.3	2.208	6.4	0.6062
MMT/PVP (70/30)	0.1881	56.6	0.4591	35.9	2.114	7.5	0.5900
MMT/PVP (60/40)	0.1908	52.4	0.4633	37.8	2.006	9.8	0.5708
MMT/PVP (50/50)	0.1796	50.7	0.4278	38.5	1.928	10.8	0.5562
Pd^2+^@MMT/PVP (80/20)	0.1861	63.2	0.4364	30.8	2.265	6.0	0.6158
Pd^0^@MMT/PVP (80/20)	0.2007	55.6	0.431	40.4	2.295	4.0	0.6208

**Table 2:**
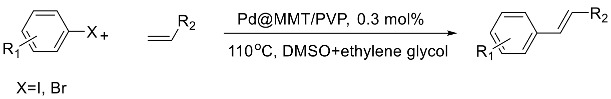
Heck reactions between aryl halides and acrylates or alkenes catalyzed with Pd@MMT/PVP catalysts. ^a^

Entry	Aryl Halides	Acrylates or Alkenes	Products	Yield ^b^
1	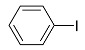	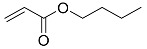	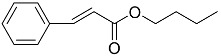	94%
2	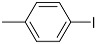	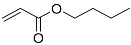	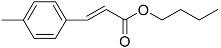	93%
3	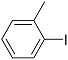	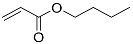	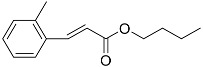	89%
4	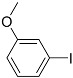	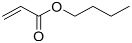	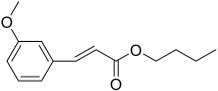	87%
5	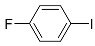	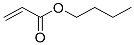	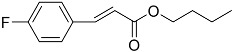	90%
6	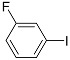	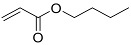	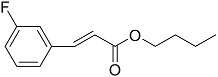	89%
7	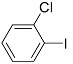	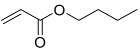	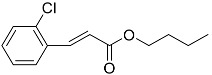	87%
8	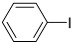	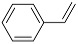	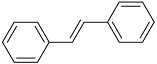	93%
9	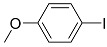	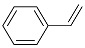	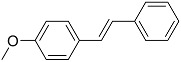	90%
10	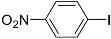	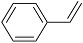	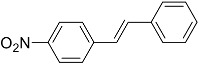	89%
11	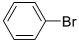	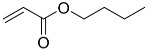	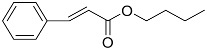	trace
12	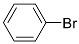	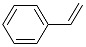	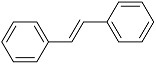	trace
13	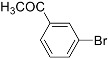	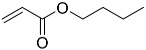	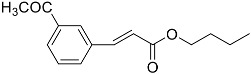	44%
14	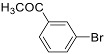	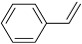	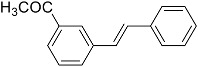	47%

^a^ Reaction conditions: 1 mmol aryl halides, 2 mmol acrylates or alkenes, 3 μmol Pd@MMT/PVP catalyst, 3 mmol CH_3_COOK, in 5 mL DMSO + 0.2 mL ethylene glycol solution, 110 °C, 5 h. ^b^ GC/MS yield.
